# A feasibility study of the use of medical clowns as hand-hygiene promoters in hospitals

**DOI:** 10.1371/journal.pone.0279361

**Published:** 2022-12-22

**Authors:** Yehuda Neumark, Adina Bar-Lev, David Barashi, Shmuel Benenson

**Affiliations:** 1 Braun School of Public Health and Community Medicine, Hebrew University of Jerusalem, Jerusalem, Israel; 2 Self-Employed, Tel Aviv-Yafo, Israel; 3 Faculty of Medicine, Department of Clinical Microbiology and Infectious Diseases, Hadassah Medical Center, Hebrew University of Jerusalem, Jerusalem, Israel; Prince Sattam Bin Abdulaziz University, College of Applied Medical Sciences, SAUDI ARABIA

## Abstract

Healthcare-acquired infections (HAI) pose vast health and economic burdens. Proper hand-hygiene is effective for reducing healthcare-acquired infections (HAI) incidence, yet staff compliance is generally low. This study assessed the feasibility, acceptability and preliminary effect of employing medical clowns to enhance hand-hygiene among physicians and nurses. Staff perception of the intervention and its impact on hand-hygiene was assessed via self-report questionnaires. Nearly 1,500 hand-hygiene compliance observations were conducted in accordance with WHO guidelines before, during and after the intervention. In each of three hospitals in Israel, two departments were selected—one in which medical clowns routinely operate and one clown-naive department. Professional medical clowns acted as hand-hygiene promoters employing humorous tactics to encourage hand-sanitizing based on the WHO "5 Moments" model. The clown appeared in each department seven times during the 2-week intervention phase. Pre-intervention hand-hygiene compliance ranged from just over 50% to 80% across hospitals and departments. Overall, about 70% of nurses (N = 132) and 80% of physicians (N = 49) felt the intervention improved personal and departmental hand-hygiene, with large inter-department variation. Pre- to post-intervention hand-hygiene compliance increased by 4% -25% (3.5–14.8 percentage points) in four departments, three of which had low baseline compliance levels. Results of this feasibility study suggest that employing medical clowns as hand-hygiene promoters as a novel approach toward HAI prevention is feasible and welcome by hospital staff.

## Introduction

Proper hand-hygiene (H-H) is considered the single most effective and cost-effective measure for reducing the incidence of hospital acquired infections (HAI) [1]. Yet, compliance remains suboptimal in hospitals the world over [2–6]. As elsewhere, Israeli hospitals still struggle with low H-H compliance [7–10].

The science of H-H progresses and various strategies to reduce the incidence of HAI and cross-transmission of pathogens have been tried [11]. H-H improvement initiatives (e.g., online training modules, visible reminders, hand-hygiene champions from among the medical staff, and empowering patients to encourage hand-washing among healthcare providers) demonstrate positive results [12–17]. However, behavior changes, in terms of sustained H-H compliance, usually require continuous reinforcement [18,19].

The limited and variable success to motivate healthcare worker compliance with hand-cleansing to date, suggests that modifying hand-hygiene behavior is a complex task that requires addressing health-related behavior influences and conditions at the intrapersonal (individual), interpersonal, institutional and community factors. Entertainment-education is a social communication strategy that seeks to capture the attention of the intended audience, often through humor, in order to deliver educational messages or bring about behavioral change [20,21]. This strategy has been used to deliver public health messages on a range of topics, including reproductive health, family planning and HIV prevention [21]. Humor interventions have been shown to enhance coping efficacy and enthusiasm for work among health care providers [22,23], possibly by reducing negative affect [24].

Education-entertainment videos aimed at encouraging H-H among hospital personnel have been produced by numerous hospitals in the USA (e.g., Somerset Medical Center, NJ: https://www.youtube.com/watch?v=UxvJDzPkxrE; Baptist Health, Jacksonville, Florida: https://www.youtube.com/watch?v=gdK6JhBLhCw), Canada (Health Sciences Centre, Winnipeg: https://www.youtube.com/watch?v=Gn-Do0k2prU), Australia (Cabrini Health Ltd., Victoria: https://www.youtube.com/watch?v=a5ZD5qyqkfQ; AlfredHealth, Victoria: https://www.youtube.com/watch?v=G6z5-RikOsg), Europe (Hôpitaux Universitaires Geneva: https://www.youtube.com/watch?v=xkdX0gjC6UE&t=110s) and Israel (Shaare Zedek Medical Center, Jerusalem: https://www.israel21c.org/israeli-hospitals. (A few of these videos used medical clowns (MCs) to convey the H-H messages (https://www.youtube.com/watch?v=9iCZeW_Jt4A; https://www.youtube.com/watch?v=1D8xSiOoK1g; https://www.youtube.com/watch?v=6sqG5v3W6D8). Content analyses of H-H edutainment videos reveal a need for better information inaccuracy and alignment with best practice principles [25,26]. None of the videos, however, were accompanied by an evaluation of the impact of the video.

### Medical/Therapeutic clowning

The origin of modern hospital clowning is most often ascribed to Michael Christensen’s Big Apple Circus Clown Care project in New York in the mid-1980s [27], although it seems that the healing effects of medial/therapeutic clowns were known to physicians in ancient Greece [28]. The goal of therapeutic clowning is to "provide a humor-based distraction to improve hospitalized pediatric patients’ moods and reduce their anxiety" [27,29,30]. In the past two decades, though, therapeutic clowning has developed into a popular practice worldwide that is no longer limited to children, and MCs can be seen in a range of treatment settings engaging with persons of all ages [23,31–39]. Studies in Israel [40–47] where the "red nose" of medical clowns is commonly seen in most hospitals in the country, and elsewhere [27,28,30,48–51], point to the positive effect and importance of MCs also from the perspective of the health care professionals [30,37,52–55].

While MCs are trained in proper hygiene practices including correct hand-sanitizing procedures, no study of MCs serving as promoters of hand-hygiene has been reported in the professional/scientific literature.

Given the novel H-H role for MCs, this study focused primarily on process measures with an aim to assess the feasibility, acceptability and preliminary effect of employing MCs to enhance H-H compliance among hospital physicians and nurses. Pre- and post-intervention outcome data were also collected as part of this non-randomised feasibility study although this was not a primary outcome of the study. Our process was guided by the Template for Intervention Description and Replication (TIDieR) checklist [56].

## Methods

### Setting

This study was undertaken in three hospitals in Israel in which MCs (from the ‘Dream Doctors’ organization) are employed. The hospitals were purposively selected (in consultation with the senior MC of the Hadassah University Hospital, DB, 3^rd^ author of this report) based on their a-priori greater likelihood of participation. In each hospital, two medical departments were selected–one in which professional MCs routinely appear and are familiar to the staff (pediatrics), and one clown-naive department (internal medicine).

### Intervention

The participating MCs received 12 hours of practical and theoretical training that included information (from SB–last author of this report) about HAI, the utility of hand-hygiene in preventing HAI, and the WHO 5 Moments, and the H-H messages to be conveyed. The MCs were also introduced to social cognitive community-level models that attempt to explain human behavior, such as the Theory of Ecological Perspective (also referred to as the Ecological Model of Behavioral Change) and other relevant behavior change models [1,16,57]. With DB, the trainees conducted simulations for the purpose of constructing a theatrical activity aimed at conveying the H-H messages. The theatrical activities were illustrated using clown language skills and techniques including attention-grabbing looks and silhouettes, rhythm-styles, acting, message-conveying text and language, props and accessories, making humor-based contact and a H-H parody.

The H-H messaging was based on the WHO 5 Moments of hand-hygiene approach [4]. This approach recommends that healthcare workers clean their hands before and after patient contact, after touching patient surroundings, before performing clean/aseptic procedures and after body fluid exposure. Proper hand hygiene practices were demonstrated and practiced in simulation sessions.

Each MC was encouraged to develop his/her own personas for the task. For example, one clown played the part of a waitress who presented the "5 Moment Menu" and offered a bottle of alcohol-based hand rubbing (ABHR) solution (hand-sanitizer) on a serving tray (Photo 1). Results of the pre-study trial suggested that the clowns should appear in different costumes/personas throughout the intervention so as to enhance the novelty.

Photo 1. Medical Clown David "Dush" Barashi as a hand-hygiene promoting waitress offering hospital staff alcohol-based hand-sanitizer.

In each department, the MC acted as a hand-hygiene promoter employing humorous tactics to encourage hand-sanitizing among hospital personnel. The clown appeared in the department a total of seven times during the 2-week intervention phase, each time for 90 minutes. These appearances were scheduled at different times of the day in order to maximize the number of staff with whom he/she engaged. While in the department, the MC engaged nurses and physicians whom he/she encountered in the corridors and in other public spaces including the public space of patients’ rooms (but not within the patient’s private space). In their routine clowning work, the MCs regularly engage with all cadres of department staff including orderlies, cleaners, and secretaries and other administrative personnel. For this feasibility study, however, the target audience was nurses and physicians.

### Data collection

Three weeks post-intervention, self-completion anonymous questionnaires were distributed (online or in person) to assess nurses’ and physicians’ personal adherence to hand-rubbing with ABHR at work (to a large extent, to a moderate extent, to a small extent, not at all), familiarity with the WHO 5 Moments (yes/no), and their perceptions regarding the MC’s intervention in their department. Specifically, awareness of the MC in the role of H-H promoter (yes/no), clarity of the purpose of the MC’s appearance (yes/no), influence of the MC on personal H-H practice and on departmental H-H practice (to a large / moderate / small extent, not at all). The respondent’s age, profession (nurse/physician), professional seniority (years working in the profession) and age were also asked. The post-intervention questionnaire was developed for the purpose of this research and was based on the questionnaire used by Ilan and colleagues [58]. The survey response rate is not available (see Limitations below).

Observations of hand hygiene compliance of nurses and physicians were performed via the use of a standardized and validated instrument and in accordance with WHO hand hygiene “Five Moments for Hand Hygiene Guidelines” [4]. Such observations are routinely carried out by the Infection Control Unit nurses of each hospital. The number of observations performed in the participating departments was increased for the purposes of this study.

To carry out an H-H observation, the observer enters the department and unobtrusively observes a randomly selected nurse or physician until one of the five H-H Moments occurs. The observer then records whether or not the anonymous person was compliant with H-H regulations using the standardized and validated form issued by the Ministry of Health of Israel and used in all hospitals. The observations were performed at different times of the day and on different days of the week during the pre-intervention phase (1–5 weeks), the intervention phase (2 weeks), and 2–6 weeks post-intervention.

### Design

**Fig 1:**
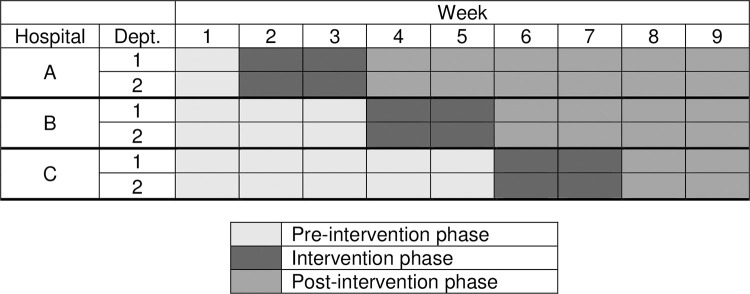
Description of the stepped-wedge intervention schedule.

Timing of the start of the intervention and the duration of the pre-intervention and post-intervention phase was planned in accordance with the stepped-wedge study design. The stepped-wedge approach is a relatively new research design that is being employed with increasing frequency in the evaluation of interventions and service delivery including hand hygiene [59–61]. In a conventional parallel-cluster design, the clusters are assigned to either the intervention or control arm at the start of the study and remain in that arm throughout the study. The stepped-wedge is an extension of the parallel-cluster study with a baseline period design, and all clusters sequentially, and at regular intervals, crossover from being a "control cluster" to an "intervention cluster" (but not simultaneously, hence the "stepped wedge"). Observations continue throughout the study, so that each participating cluster contributes pre- and post-intervention observations (Fig 1). The convenience of implementing an intervention in phases and the ethical benefit of providing the intervention to all clusters are the main reasons for employing a stepped-wedge design [62].

### Data analysis

#### Post-intervention survey

Percent frequencies and crosstab procedures were used to describe the responses of the nurses and physicians. Chi-square tests were used to examine bivariate associations between familiarity with the WHO 5 Moments, adherence to H-H guidelines, awareness of the MC as H-H promoter, clarity of the MC’s H-H messages, and influence of the intervention on personal and departmental H-H, and profession, hospital and department.

#### H-H observations

The H-H compliance measure was calculated as the number of instances in which the nurse/physician practiced H-H in accordance with guidelines divided by the total number of instances that occurred [4]. Mean compliance was then calculated for each department and hospital at each of the three study phases (before, during and after the intervention). Inter-hospital and inter-department differences in baseline H-H compliance, and changes across the phases, were tested with one-way Anova.

Statistical significance level was set at p<0.05. The data were analyzed using IBM® SPSS® Statistics 27.0.

It should be noted that as a feasibility study, the study was not powered to assess effectiveness of the intervention on H-H compliance, and sample size / power calculations were not performed.

*The IRB-Helsinki Committee of the Hadassah University Hospital waived the need for IRB approval and for informed consent from the hand-hygiene observees*. *Respondents to the post-intervention survey indicated implied agreement to participate by completing the questionnaire*, *which included the following statement in the instructions*: *“By completing the questionnaire*, *you express your consent to participate in the study”*.

## Results

### Post-intervention survey

A total of 190 post-intervention questionnaires were collected from the six departments– 123 from nurses, 49 from physicians and 18 respondents who did not indicate their profession. Nurses and physicians, had, on average, 10.4 (SD = 9.6) and 6.3 (SD = 8.9) years of work experience, respectively.

As seen in Table 1, all the nurses and nearly all the physicians who responded reported that they were familiar with the WHO’s "5 Moments model" of hand-hygiene. Nearly all nurses and physicians reported that they strictly abide by H-H guidelines.

**Table 1:** Responses to the post-intervention survey about hand-hygiene (H-H) and the medical clown (MC) intervention.

	Nurses (N = 132)	Physicians (N = 49)
Familiar with the WHO 5 Moments	100%	92%
Personally adhere to H-H guidelines	99%	92%
Aware of MC as H-H promoter	60%	35%
Understood MC’s H-H message*	82%	77%
MC’s intervention influenced personal H-H*	68%	81%
MC’s intervention influenced departmental H-H*	60%	51%

* Among those who were aware of the MC’s presence as a H-H promoter.

Overall, 60% of the nurses and 35% of the physicians reported that they had noticed the presence of the MC in the role of H-H promoter (χ^2^ = 10.9, p<0.01). Awareness of the MC intervention differed significantly across hospitals– 35%, 48%, 69% (χ^2^ = 16.4, p<0.01) and departments–ranging from 28% to 80% (χ^2^ = 33.9, p<0.0001).

Amongst those who noticed the MC, 82% of the nurses and 77% of the physicians indicated that the point of the clown’s messages was clear. Regarding the clarity of the messages, considerable inter-hospital variation– 68%, 71% and 92% (χ^2^ = 8.9, p<0.05) and inter-department variation– 63%-95% (p>0.05), was noted.

Two-thirds (68%) of nurses and 81% of physicians who noticed the MC reported that the intervention made a large/moderate impact on their personal H-H, and 60% of the nurses and 51% of the physicians felt that the intervention largely or moderately influenced the H-H of the entire department personnel (Table 1). Across departments, the proportion who reported personal H-H impact ranged from 43% to 89% (χ^2^ = 26.3, p<0.05), and from 33% to 67% regarding impact on H-H on the departmental level (χ^2^ = 38.9, p<0.001).

### Hand-hygiene compliance

During the nine week study, 1,474 hand-hygiene opportunities (indications of hand-hygiene according to protocol guidelines) were observed in the three hospitals. Of these, 1070 were among nurses (n = 443) and 398 among physicians (n = 192); profession was unknown or unrecorded for 6 observations. On average, 2.3 observations were conducted per person.

Large inter-hospital variability was noted in H-H compliance prior to the intervention–Hospital 1: 53.8% (SD 32.0), Hospital 2: 72.6% (SD 34.6) and Hospital 3: 81.8% (SD 27.7) [F(2,240) = 6.58, p<0.01]. Differences in baseline H-H compliance across departments were also observed, with percentages ranging from 50.0–82.2%.

**Fig 2:**
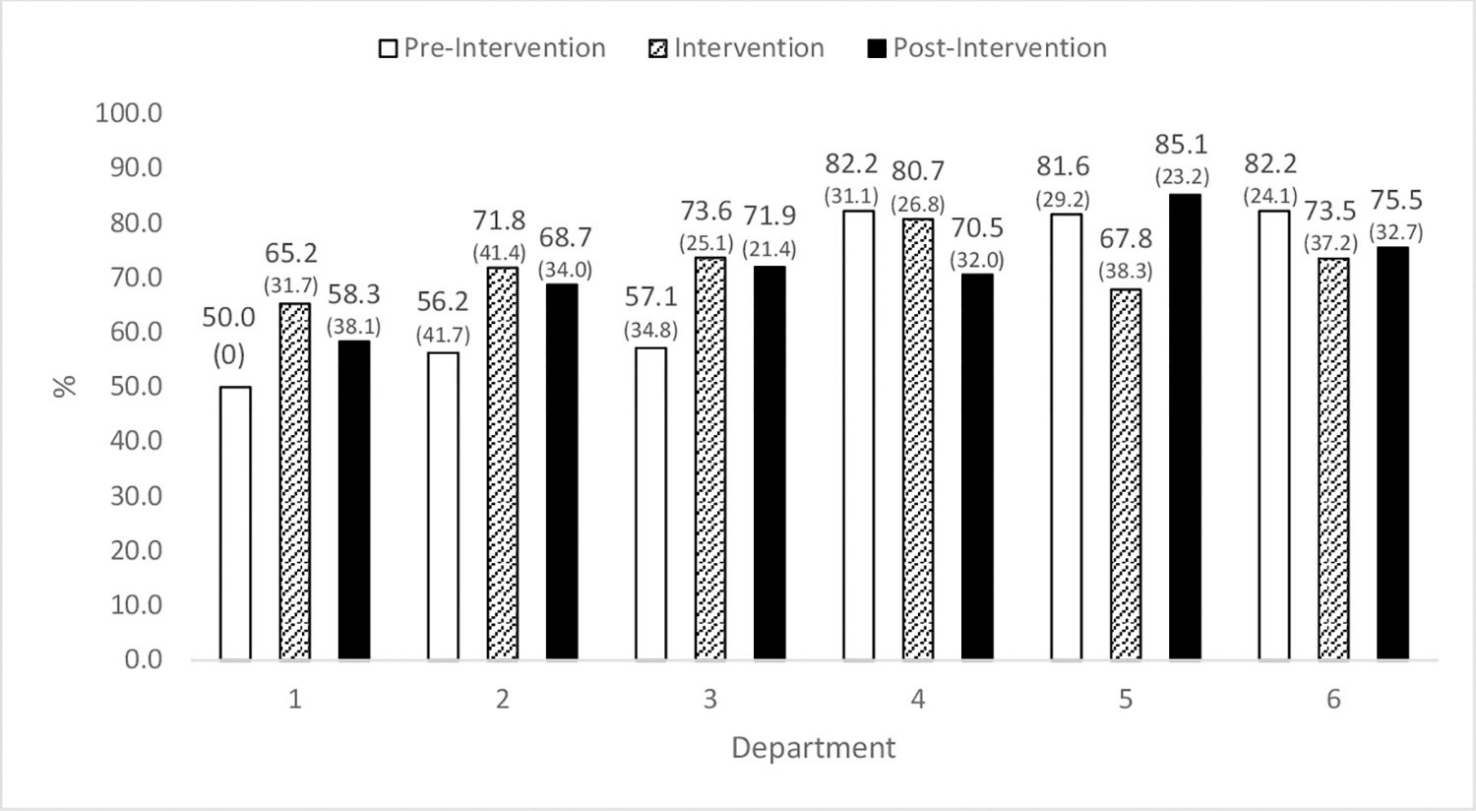
Hand-hygiene compliance–percent (SD)—by department and hospital, at baseline, during the intervention and post-intervention. Note: Hospital A: Departments 1 and 2; Hospital B: Departments 3 and 4; Hospital C: Departments 5 and 6.

In four of the six departments, a 4.3–25.9% increase (reflecting an increase in 3.5–14.8 percentage points) in H-H compliance was observed from pre-intervention to the post-intervention period. As seen in Fig 2, the increase occurred in the three departments with low baseline compliance (50–57%). In two of the three departments with high baseline compliance (82%) there was a decrease of 8.1% (11.7 percentage points) and 14.2% (6.7 points) from baseline to post-intervention. None of these changes however, were statistically significant. In four of the six departments, peak compliance was noted during the intervention phase.

There was no difference in post-intervention H-H compliance between departments in which MCs were routinely present (pediatrics) compared with departments not familiar with MCs (internal medicine).

## Discussion

The positive effect of MCs on hospital patients, particularly children, is well documented [27,29,30]. We have gone beyond this traditional role in assessing the feasibility of employing MCs as promoters of hand-hygiene among hospital medical and nursing staff. We discovered considerable pre-intervention variability in hand-hygiene compliance across departments and hospitals, ranging from 50% to 82%. These levels of compliance, based on direct observation, are similar to those found elsewhere [5,9]. In the three departments with low HH compliance at baseline, compliance increased from pre- to post-intervention but declined in the post-intervention phase, although they remained above the baseline levels. This suggests a possible relapse and the need for a longer and more sustained intervention to bring about sustained behavior change [18,19].

### Limitations

As mentioned, as a feasibility study, this study was not designed to assess the effectiveness of the clown intervention on H-H compliance. To do so would require a larger number of departments and H-H observations. We were unable to ascertain the precise number of physicians and nurses who were working in the six departments during the intervention period, and therefore we cannot calculate the response rate of the post-intervention survey. From informal and unofficial information provided from department personnel, it seems that about 2/3 of the staff returned competed questionnaires. The response rate among physicians seems to be somewhat lower than among nurses.

As mentioned above, the post-intervention questionnaire was developed for the purpose of this research and was based on the questionnaire used by Ilan and colleagues [58]. The questionnaire underwent pretesting, although no formal reliability or validity assessment was conducted. There is little reason, however, to suspect social desirability or recall bias as participation in the survey was voluntary, the questionnaire was completely anonymous and did not address sensitive topics, and the survey was conducted within a relatively short period following the intervention.

This study relied on direct observation of H-H compliance which is considered the "gold standard" for H-H monitoring, despite the near-unavoidability of observation bias [1,63]. The routine performance of H-H observations in our hospitals, and the unobtrusive (covert) manner in which the observations are conducted, help to attenuate the bias [64]. The observations were performed by Infection Control Unit nurses of each hospital as part of their routine H-H monitoring work. Although the observers were informed about the intervention, there is little reason to suspect that this knowledge influenced the validity of their observations.

As in all intervention evaluation research, there exists the possibility that an underlying temporal or secular trend (i.e., extraneous factors unrelated to the intervention may be operating in the background during the trial) may confound the intervention effect. For example, if H-H practices change over time for reasons independent of the intervention (perhaps due to the simultaneous introduction of new guidelines or sanitizing compounds or dispensers), then failure to adjust for time would result in a biased estimate of the intervention effect. The duration of the present study was short which likely precluded the possibility of such a bias arising. In larger studies of this nature, this potential bias should be addressed in the design and analysis of effectiveness trails, for example by including calendar time as a fixed effect in a generalized linear model, for example.

Our experience is that the participating hospitals and their MCs were very agreeable to participate. By piggy-backing on and augmenting the routine H-H compliance observations performed in the hospitals, we were able to carry out a large number of observations in a relatively short period. Indeed, nearly 1,500 observations were performed throughout the 9-week study. While we reported above pre- to post-intervention fluctuations in compliance in some departments, the effectiveness of the intervention was not the primary objective of this feasibility study. Not surprisingly, therefore, these changes were not statistically significant. A larger intervention trial is being planned in order to formally test the effectiveness of this novel intervention in improving H-H compliance among hospital staff. From an "ecological" perspective, and recognizing the multiple levels of influence on health behaviors, having the MC engage also with patients and with their visitors, may enhance the positive effect of the intervention on the entire department and perhaps even beyond to the broader hospital community.

More central to this feasibility study were the responses to the post-intervention questionnaires completed by nurses and physicians. A greater proportion of nurses (60%) than physicians (38%) reported having noticed the presence of the MC in the department. This is not unexpected as nurses are more present on the wards than physicians. The large inter-hospital and inter-departmental variation in the proportion of respondents who reported having noticed the MC is a key finding of this work, as it points to the centrality of the clown’s persona and particularly the level of engagement in the potential effectiveness of this intervention. The MC makes him/herself quite noticeable, and those who did not notice the MC probably did not work on the days/times when the clown was present in the department. In light of the results of the pilot intervention that suggested that a more intensive presence of the MC was needed, we increased the number of appearances the MC made in each department to seven over a two-week period. It is possible, however, that an even more intensive intervention protocol is required to ensure that the presence of the MC is noticed, particularly by physicians. The intensity of the intervention was limited by the time the MCs had to devote to this project without detracting from their routine patient support tasks, as per our a-priori agreement with the hospital administration.

Reassuringly, a high proportion (68–82%) of nurses and physicians who had noticed the MC indicated that they understood the point of the clown’s messages, and moreover, felt that the clown had influenced their own hand-hygiene and that of the entire department personnel. This supports the notion that this novel intervention may have a positive influence on hand-hygiene among those exposed to the MC. We cannot predict the duration of the behavioral modification, and regular booster clown interventions should probably be integrated into the planning of an intervention of this type.

Results of this feasibility study suggest that employing MCs in the novel role of hand-hygiene promoters may be feasible and welcome by hospital staff. Institutional and departmental permission to carry out the intervention was readily obtained from the three participating hospitals. These hospitals are medical clown-friendly and are classified as "advanced" in terms of WHO hand-hygiene practices, factors that no doubt contributed to obtaining permission. It is likely that hospitals unfamiliar with MCs and/or that are less hand-hygiene-minded, may be less enthusiastic initially about consenting to a clown-based intervention, despite the low cost and relative ease of implementation. In such situations, it will be necessary to invest the time to introduce the concept and practice of medical-clowning to the senior hospital and departmental administration. It is worth noting that another hospital that was approached to take part in this project declined to participate due to the heavy workload of the infection control unit. This is a barrier that may present itself in other settings as well. In this instance our attempts to explain that the proposed intervention and the greater number of H-H observations that would be performed (in this case, at the expense of the research project), may in the long run reduce the department’s workload, were unsuccessful in convincing the infection control unit to participate.

In summary, an intervention using medical clowns to improve hand-hygiene among hospital personnel seems to be implementable and acceptable, and may be effective in promoting hand-hygiene among healthcare workers. A major advantage of the intervention is that it is based largely on resources available in most of the hospitals in Israel. Medical clowns are employed in numerous hospitals and can be trained as health promoters to improve healthcare workers hand-hygiene compliance. Findings of this preliminary study suggest that the intervention must be intensive and carried out over a sustained time-period. A program-trial evaluation, including control departments/hospitals, should be undertaken to formally assess the effectiveness of this novel intervention. The possibility of extending the intervention to patients, family members and visitors, should be considered in order to raise awareness among the general population at risk to hospitals acquired infections and lower the seemingly immutable challenge of hospital-inquired infections.

## Supporting information

S1 Data Anonymized data set. (XLSX)
